# *SCFSen*: A Sensor Node for Regional Soil Carbon Flux Monitoring

**DOI:** 10.3390/s18113986

**Published:** 2018-11-16

**Authors:** Guoying Wang, Xiaoping Wu, Lufeng Mo, Jizhong Zhao

**Affiliations:** 1 School of Electronic and Information Engineering, Xi’an Jiaotong University, Xi’an 710049, China; wanggy.cs@gmail.com (G.W.); zjz@xjtu.edu.cn (J.Z.); 2 School of Information Engineering, Zhejiang A & F University, Hangzhou 311300, China; wuxipu@gmail.com

**Keywords:** wireless sensor networks, soil carbon flux measurement, spatial and temporal heterogeneity, dynamic chamber method

## Abstract

Estimation of regional soil carbon flux is very important for the study of the global carbon cycle. The spatial heterogeneity of soil respiration prevents the actual status of regional soil carbon flux from being revealed by measurements of only one or a few spatial sampling positions, which are usually used by traditional studies for the limitation of measurement instruments, so measuring in many spatial positions is very necessary. However, the existing instruments are expensive and cannot communicate with each other, which prevents them from meeting the requirement of synchronous measurements in multiple positions. Therefore, we designed and implemented an instrument for soil carbon flux measuring based on dynamic chamber method, *SCFSen*, which can measure soil carbon flux and communicate with each other to construct a sensor network. In its working stage, a *SCFSen* node measures the concentration of carbon in the chamber with an infrared carbon dioxide sensor for certain times periodically, and then the changing rate of the measurements is calculated, which can be converted to the corresponding value of soil carbon flux in the position during the short period. A wireless sensor network system using *SCFSen*s as soil carbon flux sensing nodes can carry out multi-position measurements synchronously, so as to obtain the spatial heterogeneity of soil respiration. Furthermore, the sustainability of such a wireless sensor network system makes the temporal variability of regional soil carbon flux can also be obtained. So *SCFSen* makes thorough monitoring and accurate estimation of regional soil carbon flux become more feasible.

## 1. Introduction

Soil respiration is a process of soil releasing carbon dioxide, which is produced by the oxidation of organic matter and the breath procedure of plant roots, and a little part of which is released by soil animals and chemical oxidation. The changes of the soil respiration rate reflects the sensitivity and tolerance of ecological systems subjected to environmental stress [1,2,3,4]. Soil respiration is an important index of soil quality and soil fertility and to a certain extent reflects the soil oxidation ability [5]. Soil respiration is one of the parameters for the prediction of the response of ecosystem productivity to climate change. In particular, the basic part of soil respiration reflects biological characteristics of soil and the metabolism intensity of soil material [6]. The process of soil releasing carbon dioxide to the atmosphere through soil respiration is a key ecological process leading to global climate change, which has been one of the core problems concerned in global carbon cycle researches [7,8].

Soil carbon flux, also called the intensity of soil respiration, means the rate of carbon dioxide released from soil. The measurement of multi-position soil carbon flux is important to reveal the detail distribution of the soil carbon flux in a region and the estimation of regional soil carbon flux, which can be used to explore the carbon cycle and carbon balance in the atmosphere [9,10,11]. The measurement of soil carbon flux at one or several near locations can be carried out using some existing soil carbon flux measuring systems such as the LI-8100 series of instruments manufactured by LI-COR Company (Lincoln, NE, USA) [12]. In order to monitor soil carbon flux of a wide area, the sampling should be carried out in much more positions within the area [13]. However, currently existing instruments are not very suitable for large-area, long-term and continuous monitoring of regional carbon flux of a terrestrial ecosystem. The main reasons include limited measuring positions, inconveniences for synchronized measurements and high energy consumption. Even if a LI-8150 Multiplexer, an accessory for the LI-8100, is used, maximum 16 individual chambers can be connected to LI-8100 analyzer control unit and be controlled and sampled in a field with maximum diameter of 30m, which is not completely sufficient for large-area monitoring. If multiple systems based on LI-8150 is used, besides the cost, the cooperative controlling among them is another issue what needs to be considered for synchronized measurements.

If only a few positions are chosen to carry out soil respiration measurements at different time for a short period, the estimation of the regional soil carbon flux in an area is undoubtedly inaccurate for the spatial heterogeneity and temporal variation of soil respiration [14,15]. So, to correctly measure and accurately estimate the soil carbon flux of a given region, there are some requirements that should be met and can’t be easily and well met using currently existing devices and traditional methods.

(1) Measurements should be taken in multiple positions to dominate the whole monitored region. For the spatial heterogeneity of soil respiration, the sampling positions should be sufficient to express the region based on the spatial correlation.
(2) Measurements should be carried out and the data should be kept on gathering for a relatively long time. For the temporal variation of soil respiration, each measurement only denotes the soil respiration situation at that moment. We need measure the soil carbon flux at different time in each sampling position in the monitored region.
(3) Measurements in different positions should be able to be synchronized controlled. Simultaneous measurement results of different positions can exactly express the overall situation of the region at the same moment.

The wireless sensor network technology can meet these requirements well. A wireless sensor network consists of many sensor nodes with sensing and communicating abilities, and these nodes are deployed in the monitored area and formed an autonomous networking system [16,17]. Besides the three advantages mentioned above, a wireless sensor network has an additional convenience that data can be transferred to remote central server for real-time displaying, storing, processing and analyzing [18].

The wireless sensor network technology has been widely used in environmental or ecological applications, such as precision agriculture [19,20], wild environment monitoring [21], canopy closure sustainable estimation [16], volcano monitoring [22], wildlife monitoring [23], marine environment monitoring [24] and so on. In previous works, the wireless sensor network technology has already been used for the measurement or monitoring about soil. For example, soil property monitoring [25], soil parameters estimation [26], soil moisture [27,28], soil slopes [29], wireless communication in soil [30] and so on.

In order to apply wireless sensor network technology to regional soil carbon flux monitoring, a basic requirement that should be met firstly is the corresponding sensor nodes. These nodes should be able to not only measure soil carbon flux but also support communication protocol of wireless sensor networks at least. However, there is no such device available.

**Figure 1:**
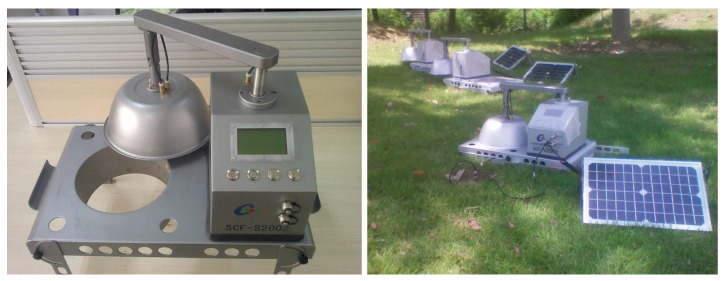
*SCFSen*.

This is just the main motivation of this paper. The theory, designing and implementation of a kind of soil carbon flux measuring instrument, *SCFSen*, which can serve as sensor nodes, are illustrated in detail in this paper. The exterior of *SCFSen* is shown in Figure 1.

The key contributions of this paper are as follows.

(1) The designing and implementation of *SCFSen*, a new instrument for soil carbon flux measurement, are introduced. From the aspect of functionality, *SCFSen* can support WSN communication besides soil carbon flux measurement, which make it suitable for regional soil carbon flux monitoring by constructing a sensor network.
(2) The energy consumption of *SCFSen* is analyzed and compared with that of LI-8100. The working time of *SCFSen* can be about 23 days if three consecutive measurements are taken per hour, which is more than two times longer than that of LI-8100 for the same measurement task. *SCFSen* can keep working for about 55 days if it is set to take one measurement per hour. Furthermore, *SCFSen* can be recharged by a solar panel in practice, which leads to much longer working time and the possibility for sustainable monitoring.
(3) A grouped calibration method for *SCFSen* nodes is proposed and tested. After calibration, the mean relative errors of *SCFSen* nodes can be reduced from over 15% to about 6%, taking the result of LI-8100 as ground truths. The difference between the results from two different instruments is reasonable.

The remaining parts of this paper are organized as following. The model of soil carbon flux measurement used in *SCFSen* is demonstrated in Section 2. The detail of designing and implementation about the mechanical structure, the electrical structure, and the analysis of energy consumption of *SCFSen* are introduced in Section 3. The calibration method of *SCFSen* are given in Section 4. Experiments of *SCFSen* are given and analyzed in Section 5. At last, the paper is concluded by Section 6.

## 2. Method of Soil Carbon Flux Measurement

### 2.1. Model of Dynamic Chamber Method

Soil carbon flux can be measured using chamber methods, including static chamber method and dynamic chamber method [31,32]. The static chamber method means measuring the carbon dioxide contents in the chamber just before and after a period and calculating the carbon flux according to the difference between the two measurements, which are usually carried out manually with subsequent offline analysis of gas chromatograph. The time needed for this method is a little long, usually from dozens of minutes to several hours, and the result is coarse – grained and its accuracy is relatively low.

Dynamic chamber method is considered as an ideal soil respiration measurement method [33]. The dynamic measurement method can get soil CO${}_{2}$ emission values more accurately than the static measurement method, and is more suitable for the determination of the rate of CO${}_{2}$ emission for a period of time. As to the dynamic chamber method, the rate of CO${}_{2}$ diffusion into the air is estimated though the measurement of the changing rate of CO${}_{2}$ concentration in the chamber, which is carried out in an in situ way.

**Figure 2:**
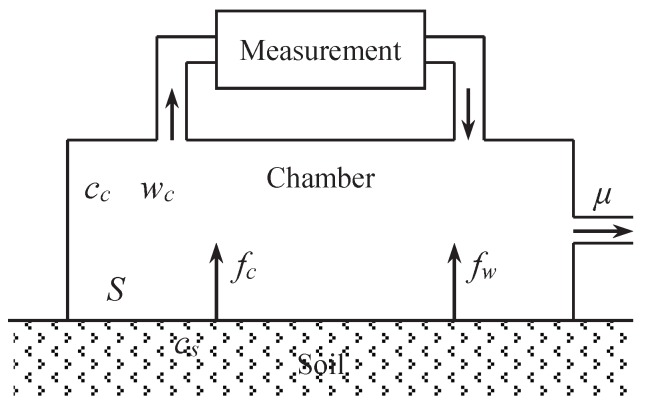
Measuring model of dynamic chamber method. *S*: area of soil surface covered by the chamber; $c_{c}$: mole fraction of CO${}_{2}$ in the chamber; $w_{c}$: mole fraction of water in the chamber, $f_{c}$: emission rate of carbon dioxide from the soil, $f_{w}$: emission rate of water vapor from the soil, $c_{s}$: mole fraction of CO${}_{2}$ in soil, μ: rate of gas emission for the balance.

The structure of dynamic chamber method is shown in Figure 2. During the measurement, a chamber is covered on the sampling spot. There is a loop back tube with the chamber, one end of which is for the extraction of gas in the chamber and feeding to the module of measurement of concentration of carbon dioxide in the gas, and the other end is for the return of gas after measurements. During the measuring procedure, the concentration of carbon dioxide in sampled gas from the chamber is measured for multiple times periodically. In addition, then the amount of soil releasing or absorbing carbon dioxide can be calculated according to the changing rate of the concentration of carbon dioxide in the chamber based on the principle of air diffusion and convection between soil and atmosphere. To keep the balance of pressures between inside and outside of the chamber, there is the third hole with the chamber.

In general, the soil layer contains a large number of microorganisms, and continuously releases CO${}_{2}$ into the air. In addition, there is some water content in the soil, which is being evaporated into the air all the time. Assuming that the total volume of the chamber and the tube in the measurement system is *v* m${}^{3}$, the area of soil surface covered by the chamber is *S* m${}^{2}$, the emission rate of carbon dioxide is $f_{c}$ mol·m${}^{-2}$·s${}^{-1}$, and the emission rate of water vapor from the soil is $f_{w}$ mol·m${}^{-2}$·s${}^{-1}$.

The gas in the chamber is mainly composed of dry air, water vapor and CO${}_{2}$. Let ρ denote the total gas concentration and its unit is mol·m${}^{-3}$. So we get Equation (1).

(1) $$\rho =\rho_{d}+\rho_{c}+\rho_{w}$$

In Equation (1), $\rho_{d}$, $\rho_{c}$ and $\rho_{w}$ mean the concentrations of dry air, CO${}_{2}$, and water vapor in the chamber, respectively, and their units are all mol·m${}^{-3}$.

Let $c_{s}$ denote the mole fraction of CO${}_{2}$ in soil whose unit is mol·mol${}^{-1}$, $c_{c}$ and $w_{c}$ denote the mole fraction of CO${}_{2}$ and water vapor in the chamber, respectively, whose unit are all mol·mol${}^{-1}$, and μ denotes the rate of gas emission for the balance whose unit is mol·s${}^{-1}$. As to $c_{c}$ and $w_{c}$, we can get Equations (2) and (3).

(2) $$\rho_{w}=\rho \cdot w_{c}$$

(3) $$\rho_{c}=\rho \cdot c_{c}$$

According to the principle of gas flow balance, we can get Equations (4) and (5).

(4) $$v\cdot \frac{\partial \rho_{w}}{\partial t}=S\cdot f_{w}-\mu \cdot w_{c}$$

(5) $$v\cdot \frac{\partial \rho_{c}}{\partial t}=S\cdot f_{c}-\mu \cdot c_{c}$$

As the content of water vapor is much higher than that of CO${}_{2}$ in the chamber, we can get Equation (6).

(6) $$\mu \approx S\cdot f_{w}$$

According to Equations (2), (4) and (6), we can get Equation (7).

(7) $$\mu \approx \frac{v\cdot \rho }{1-w_{c}}\cdot \frac{\partial w_{c}}{\partial t}$$

From Equations (3), (5) and (7), we can get Equation (8).

(8) $$f_{c}=\frac{v\cdot \rho }{S}\cdot \left(\frac{\partial c_{c}}{\partial t}+\frac{c_{c}}{1-w_{c}}\cdot \frac{\partial w_{c}}{\partial t}\right)$$

Let $c_{c}^{\prime }=c_{c}/\left(1-w_{c}\right)$, we can get Equation (9).

(9) $$\left(1-w_{c}\right)\cdot \frac{\partial c_{c}^{\prime }}{\partial t}=\frac{\partial c_{c}}{\partial t}+\frac{c_{c}}{1-w_{c}}\cdot \frac{\partial w_{c}}{\partial t}$$

According to Equations (8) and (9) can be transformed to Equation (10).

(10) $$f_{c}=\frac{v\cdot \rho }{S}\cdot \left(1-w_{c}\right)\cdot \frac{\partial c_{c}^{\prime }}{\partial t}$$

According to the ideal gas law, we have $\rho =p_{0}/\left(R\cdot T\right)$, in which $p_{0}$ stands for the pressure of gas and its unit is Pa, R=8.31441 means the gas constant and its unit is Pa·m${}^{3}$·K${}^{-1}\cdot$mol${}^{-1}$, and *T* denotes the absolute temperature and its unit is *K*, so Equation (10) can be rewritten as Equation (11).

(11) $$f_{c}=\frac{v\cdot p_{0}}{R\cdot S\cdot T}\cdot \left(1-w_{c}\right)\cdot \frac{\partial c_{c}^{\prime }}{\partial t}$$

As Equation (11) shows, we can see that $f_{c}$ can be calculated when $p_{0}$, *T*, $w_{c}$ and $\frac{\partial c_{c}^{\prime }}{\partial t}$ are measured, as *v* and *S* are constant once the chamber is determined. $p_{0}$, *T* and $w_{c}$ can be measured directly using corresponding sensors, so the remaining challenging issue is the measurement of changing rate of carbon dioxide concentration in the chamber.

### 2.2. Measurement of Changing Rate of Carbon Dioxide Concentration in the Chamber

According to dynamic chamber method, the carbon dioxide emission rate in per unit area of soil is calculated through measuring and analyzing the variation of the carbon dioxide concentration in the chamber. As the concentration of carbon dioxide in the chamber can be measured directly, the changing rate can be calculated by fitting some temporally adjacent measurements of the concentration.

However, the changing rate of concentration of carbon dioxide in the chamber is not a constant value during a measuring procedure, because the releasing rate of carbon dioxide from soil is affected by the difference between carbon dioxide concentration in soil and that in the chamber. The carbon dioxide concentration in the chamber is keep increasing along with the emission of carbon dioxide from soil to chamber, which makes the difference become smaller and smaller.

Now we analyze the changing pattern of concentration of carbon dioxide in the chamber along with time to find a proper method for the calculation of the changing rate. As the concentration of CO${}_{2}$ released from the soil to the chamber is determined by the difference between the CO${}_{2}$ concentrations in them, so there is a relationship between $f_{c}$ and $\left(c_{s}-c_{c}\right)$ as Equation (12).

(12) $$f_{c}=\rho \cdot g\cdot \left(c_{s}-c_{c}\right)$$

The *g* in Equation (12) denotes the gas conductive coefficient of CO${}_{2}$ whose unit is m·s${}^{-1}$. Combine the two Equations (10) and (12), we can get Equation (13).

(13) $$\frac{\partial c_{c}^{\prime }}{\partial t}+\frac{S\cdot g}{v}\cdot c_{c}^{\prime }=\frac{S\cdot g}{v}\cdot c_{s}^{\prime }$$

In Equation (13), $c_{s}^{\prime }=c_{s}/\left(1-w_{c}\right)$.

Assuming that the initial value of $c_{c}^{\prime }$ is $c_{c}^{\prime }\left(0\right)$, solve the differential Equation (13), we can get Equation (14).

(14) $$c_{c}^{\prime }\left(t\right)=c_{s}^{\prime }+\left[c_{c}^{\prime }\left(0\right)-c_{s}^{\prime }\right]\cdot e^{-\alpha \cdot t}$$

In Equation (14), α=g·S/v. Differentiate the both side of Equation (14), we get Equation (15).

(15) $$v\frac{\partial c_{c}^{\prime }}{\partial t}=\alpha \cdot \left[c_{s}^{\prime }-c_{c}^{\prime }\left(0\right)\right]\cdot e^{-\alpha \cdot t}$$

As to a determined implementation, α can be regard as a constant value because *g*, *S* and *v* are all constants during a measuring procedure. In addition, the value of $c_{s}^{\prime }-c_{c}^{\prime }\left(0\right)$ can also be regard as a constant during a measuring procedure because $c_{c}^{\prime }\left(0\right)$ is a constant and $c_{s}^{\prime }$ does not vary sharply. As a result, the changing rate of concentration of carbon dioxide in the chamber, $c_{c}$, is mainly affected by *t* in a measuring procedure.

**Figure 3:**
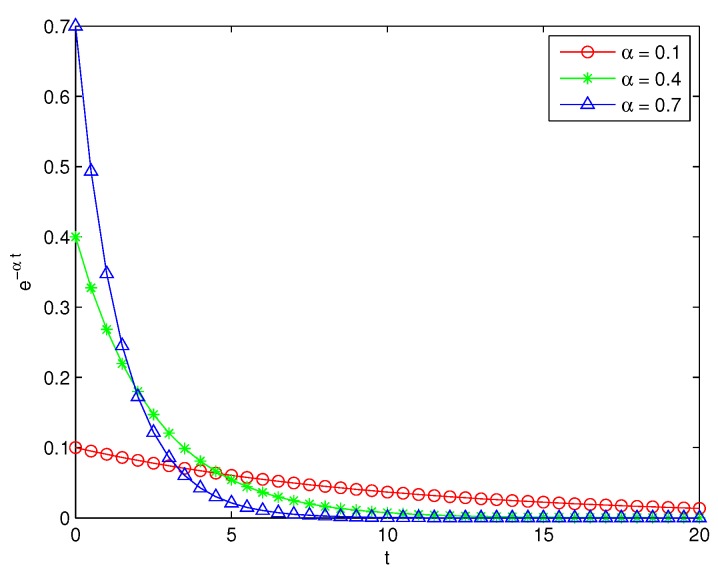
Trends of $\alpha \;\times \;e^{-\alpha \cdot t}$ for different α values.

As we know, the curves of $\alpha \times e^{-\alpha \cdot t}$ are as Figure 3, in which the values of α for the three curves are 0.1, 0.4 and 0.7, respectively. Here the three values of α are just for the illustration of trends of the curves. From the comparison of the three curves, we can see that the bigger α is, the more sharply the values of longitudinal coordinates change. This can be explained by that the bigger *S* is or the smaller *v* is, the more easily the air in the chamber is affected by the soil respiration, and the more quickly the changing rate of carbon dioxide concentration in the chamber varies.

There is no chamber in a real environment and the concentration of carbon dioxide above soil do not change sharply. The initial changing rate of carbon dioxide in the chamber should be adopted for the calculation of soil carbon flux, because the air in the chamber in the beginning phase is closer to real environment without a chamber than that in the later phase.

To calculate the changing rate of carbon dioxide concentration in the chamber, multiple measurements of carbon dioxide concentration during a period should be linearly fitted. There is a confliction between the duration of time and the accuracy of the changing rate calculation. If the duration of time is too short, the differences among measurements during this period are not obvious and the changing rate cannot be fitted well. If it is too long, the fitting can be easily done, but the accuracy may decrease because the measurements is not linear for the decent of changing rate along with time.

As a trade-off based on experiments, we adopt the periodical 60 measurements in the beginning 3 min in every procedure of soil carbon flux measurement as the source data to calculate the changing rate of concentration of carbon dioxide, on the basis of which the value of $\frac{\partial c_{c}^{\prime }}{\partial t}$ in Equation (11) can be calculated.

### 2.3. Calculation of Soil Carbon Flux

Based on the periodical measurements of carbon dioxide concentration in the chamber, the changing rate of concentration of carbon dioxide is get. In addition, then, the values of the measurements can be converted to the standard condition and the soil carbon flux value of unit area and unit time under the standard condition, whose unit is mol ·m${}^{-2}$·s${}^{-1}$, can be obtained according to the ideal gas law.

The method for the calculation of soil carbon flux is described as follows.

(1) Determine the total volume of the chamber, *v* (m${}^{3}$), and the soil surface area in the chamber, *S* (m${}^{2}$). Measure the initial pressure $p_{0}$ (Pa), the initial temperature, $T_{0}$ (${}^{\circ }$C) in the chamber.

(2) The relative humidity, the ratio between the mole fraction of water vapor in air and the mole fraction of saturated water vapor under the same temperature and pressure, are measured in *SCFSen*. Assume that the value of relative humidity is φ, the mole fraction of saturated steam under the same temperature $T_{0}$ and pressure $p_{0}$ is $w_{0}$, which can be get by looking up the saturated steam table. Therefore, the mole fraction of water vapor in the air can be calculated using Equation (16).

(16) $$w_{c}=\varphi \cdot w_{0}$$

(3) Determine the changing rate of the concentration of carbon dioxide in the chamber by fitting the sampling values of that measured periodically in the first three minutes, which is the value of $\frac{\partial c_{c}^{\prime }}{\partial t}$ and the unit is ppm·s${}^{-1}$.

(4) According to Equations (11) and (16), we can reach Equation (17), which is the method of calculating soil carbon flux used in *SCFSen*, whose unit is µmol·m${}^{-2}$·s${}^{-1}$

(17) $$f_{c}=\frac{v\cdot p_{0}}{R\cdot S\cdot (T_{0}+273.15)}\cdot \left(1-\varphi \cdot w_{0}\right)\cdot \frac{\partial c_{c}^{\prime }}{\partial t}$$

## 3. The Design and Implementation of *SCFSen*

In this section, the mechanical structure, the electrical structure and energy consumption analysis of *SCFSen* are introduced one by one.

### 3.1. The Mechanical Structure

**Figure 4:**
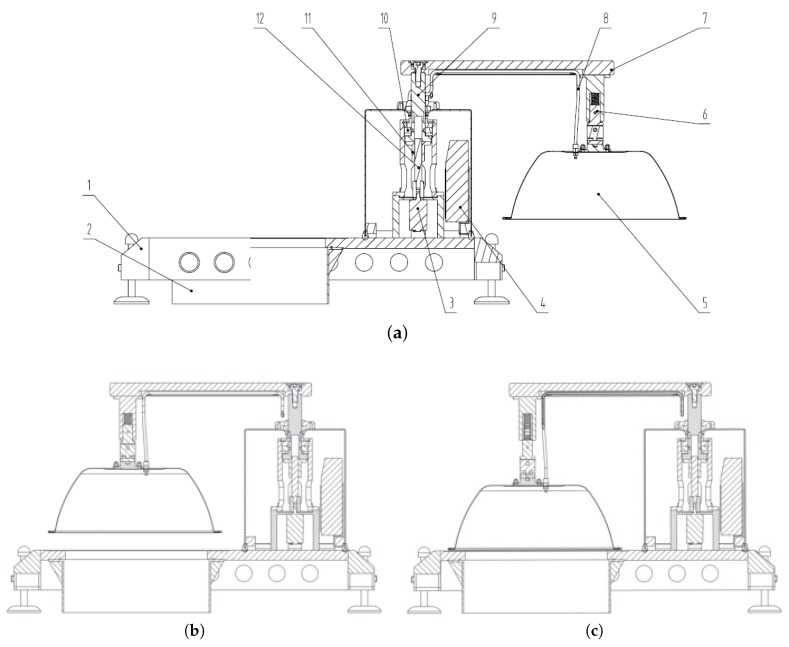
Mechanical structure of *SCFSen*. (**a**) During interludes between measurements. 1—base frame; 2—fixing ring; 3—motor; 4—controlling module; 5—chamber cover; 6—buffered connecting rod; 7—rocker arm; 8—pipe; 9—track connecting rod; 10—rocker pin; 11—sleeve; 12—motor connecting rod. (**b**) Before measurements. (**c**) During measurements.

*SCFSen* adopts the mechanical structure as Figure 4, and it can reduce the influence of measurement on soil environment. The mechanical structure consists of supporting and driving structures. The main supporting structure includes the base, the fixing ring and the measuring chamber cover. The driving structure is composed of the motor, the buffering connecting rod, the track connecting rod and the lock seat and the rocker arm. The motor is fixed on the base, and its shaft links to the motor connecting rod, the track connecting rod and the rocker arm using fasteners. The motor connecting rod is connected with the track connecting rod through a clamping sleeve, and can carry out axial relative movement. A spring is mounted with the buffering connecting rod, so as to distribute the motor force to the chamber cover evenly. The rotation and the vertical movement of the chamber cover are driven by a single motor along the specific track set by the track connecting rod.

The fixing ring and the chamber cover construct a chamber together during the measurements. When deploying a *SCFSen* instrument to a selected position, a circular narrow groove need be dug according to the size of the fixing ring, and then the bottom of the fixing ring is plugged into the groove, so as to construct a chamber when the chamber cover is covered.

After a *SCFSen* instrument is deployed, the controlling module controls the transmission structure to open or close the chamber periodically. During the interludes between measurements, the chamber is open and the cover is not above the fixing ring, as Figure 4a shows. The purpose is to let the measuring position, the field in the fixing ring, be exposed to sunlight, rain, wind and so on, so as to minimize the impacts of measurement behaviors on measurement results.

Before the instrument starts to measure, the controlling module will turn on the motor, and the motor connecting rod and the track connecting rod will do relative rotation movement. The rotation of the chamber cover is realized through the special track on the track connecting rod driving the rocker arm to rotate. As a result, the chamber cover is rotated when the rocker arm rotates. When the chamber cover is rotated to a certain position, right above the fixing ring, as Figure 4b shows, the rotation will stop.

Then the chamber cover will move along the vertical direction, until it is covered the fixing ring. The vertical movements of the chamber cover are carried out cooperatively by the motor connecting rod, the track connecting rod and the rocker pin. There is a specific track on the circumferential surface of the track connecting rod, and there is a fixed and non-connected pin embedded the track. Along with the rotation of the track connecting rod, the rocker arm and the chamber cover move downwards together under the interaction force between the pin and the track. After the motion of chamber is stopped, a measuring chamber is formed, as Figure 4c shows. Then the instrument will start to measure the soil carbon flux.

After the measuring procedure, the rocker arm drags the chamber cover moving towards the opposite direction and returns to the initial position, as Figure 4a shows.

### 3.2. The Control Circuit Structure

**Figure 5:**
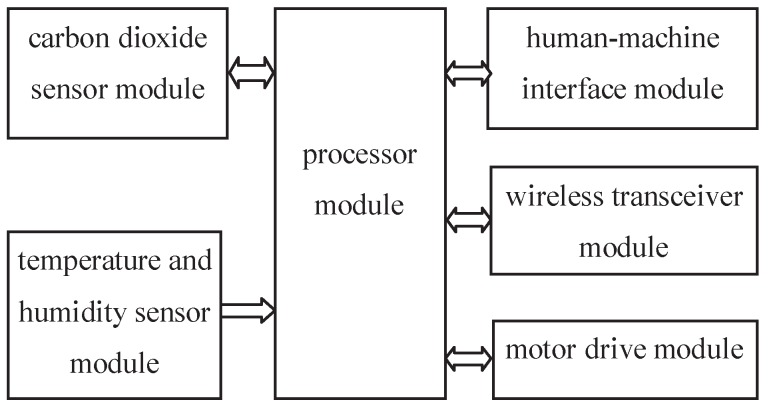
Block diagram of control circuit of *SCFSen*.

The configuration of control circuit of *SCFSen* is shown in Figure 5. It includes the processor module, the wireless transceiver module, the carbon dioxide sensor module, the temperature and humidity sensor module, the motor drive module, the human-machine interface module and the power module. The chipsets adopted for these modules of *SCFSen* are listed in Table 1.

There are different wireless communication technologies for the construction of a wireless sensor network, such as ZigBee, LoRa, WirelessHART, Z-Wave, and so on. LoRa has a transmit range of 5 km in urban areas, and up to some 15 km in rural environments, so it can be used for wide-area networks. WirelessHART is based on the highway addressable remote transducer protocol (HART). It is considered suitable to be used in industrial applications. Z-Wave is a low-power RF communications technology that is primarily designed for home automation for products such as lamp controllers.

ZigBee is a short-range IoT protocol aimed at connecting several devices in close proximity. It does not have central controller and loads are distributed evenly across the network. There are some new versions of ZigBee, such as ZigBee PRO and ZigBee Remote Control (RF4CE). These new versions have some significant advantages in complex systems offering low-power operation, high security, robustness and high scalability with high node counts and is well positioned to take advantage of wireless control and sensor networks. The latest version of ZigBee is 3.0, which is essentially the unification of the various ZigBee wireless standards into a single standard.

For the current version of *SCFSen*, we used CC2420 chip, which supports ZigBee protocol, to construct the wireless sensor network. The main reason is that *SCFSen* nodes are supposed to communicate and cooperate with the sensor nodes in GreenOrbs [16], a wireless sensor network constructed in 2009 for ecological monitoring deployed in a forest environment, as Section 5.2 shows. In the future, we will also design new versions of *SCFSen* using other communication protocols.

The processor MSP430F1611 has the characteristics of low power consumption, and can work stably under wild conditions. As mentioned above, *SCFSen* uses CC2420 chip as the wireless transceiver module, which is a kind of radio transceiver conforming to the of IEEE 802.15.4 2.4G Hz standard, which can ensure the communication efficiency and reliability with the adjacent instruments within 200 m. The processor communicates with the wireless transceiver module using the serial peripheral interface. MSP430F1611 works in the master mode, and CC2420 is in the slave mode.

The carbon dioxide sensor T6615, a kind of dual channel infrared carbon dioxide sensor, is a small and light Non-Dispersive Infra-Red (NDIR) CO_2_ sensor. NDIR is a method based on the theory of gas absorption. After the absorption of a certain gas whose concentration is to be measured, the spectral intensity of the infrared ray emitted by an infrared light source will change. According to the theory of gas absorption, the decrement of the spectral intensity is proportional to the concentration of gas, so the concentration of the gas to be measured can be calculated by measuring the decrement of the infrared ray spectral intensity. T6615 has some plug-pins which makes it very convenient to connect with other instruments. Furthermore, T6615 has several kinds of output interfaces for transmission and reading. T6615 communicates with other modules via a 19200-baud universal asynchronous receiver transmitter (UART) interface.

The digital temperature and humidity sensor SHT15, which belongs to surface mounted devices (SMD) encapsulation series, is suitable for reflow soldering. The sensing element and the signal processing circuit, integrated on a micro circuit board, output completely calibrated digital signal. SHT15 includes a capacitive polymer humidity sensitive element and a temperature measuring components made from band-gap material. These two elements are on the same chip, and are connected with a fourteen bits A/D converter and a serial interface circuit.

The motor drive module uses an H-bridge to realize the forward/reversal rotation of the motor, and the transmission mechanism drives the motion of the chamber cover. The drive motor, a DC general motor ZGA17RU877i5600, uses 12 V voltage to drive, and can output 5 r/min speed, which can meet the speed requirement of the system. When the air chamber moves downwards to the end, the electric current of the drive motor will increase sharply, according to which the closed or open state of the air chamber cover can be judged.

The power module includes a lithium ion rechargeable battery YSD-12980 with the capacity of 9800 mAh and an ultra small DC-DC buck converter chip MAX1836, a product of the Maxim company, to get the +3.3 V voltage. MAX1836 high-efficiency step-down converters is a micro power regulator, and can provide quiescent current as low as 12 µA. Its input voltage is 4.5∼24 V and its rated output voltage is +3.3 V. In order to prolong the working time of the device, a solar panel is added to charge the battery YSD-12980.

The human-machine interface module includes some buttons for the configuration of parameters such as the measuring frequency and a LCD screen, QC12864B, for the display of working state and the real-time display of parameters.

### 3.3. Energy Consumption Estimation

For the application of long-term automatic monitoring of soil carbon flux in the wild environment, low power consumption is an important issue of the control circuit design. So the sensors modules, the wireless transceiver module and the human-machine interface module are designed for low power consumption, that is to say, the power supply of these modules are switched off when they are in standby mode in order to save energy consumption. The electric current consumptions of all modules in every one hour are listed in Table 2, if measurement is carried out at intervals of one hour.

As can be calculated from the data in Table 2, the power consumption of once soil flux measurement using *SCFSen* is about 320 J if it is set to measure once every hour, because the battery is 12 V DC and its capacity is 9800 mAh. A *SCFSen* node can keep working continuously for 1323 h in theory, i.e., over 55 days, which, by and large, can meet the requirement of soil carbon flux monitoring in the wild environment. We can recharge a *SCFSen* node at intervals of almost one and a half months, and an additional solar panel can be connected to a *SCFSen* node for recharging.

For a LI-8100 system with one chamber, to get a soil carbon flux “reading” after the chamber is closed, it needs maximum 60 s for a dead band and 90 s for the observation, so total 150 s time is need. As to *SCFSen*, it takes 3 min to do the measurement, which needs more time than LI-8100, in order to get abundant raw data for the calculation of soil carbon flux.

After every measurement, LI-8100 needs a 2-min observation delay including an about 45-s purge time before next measurement. According to the LI-8100 manual, if three consecutive measurements every hour are set to take, a LI-8100 system with one chamber can keep working for 240 h (10 days). For *SCFSen*, if the same amount of measurements is required to take, it will consume about 752 J energy every hour. Because the battery *SCFSen* used is 9800 mAh and 12 V, it can keep working for 563 h (23 days) for such measurement requirement even if there is no solar panels attached.

## 4. Calibration of *SCFSen*

The readings of sensors tend to be error-prone. Due to the existence of instrumental errors of sensors, the raw measurement of a *SCFSen* node often is not completely correct. So the calibration for every *SCFSen* node before its use for the first time is very important.

### 4.1. Preliminary Experiment

**Figure 6:**
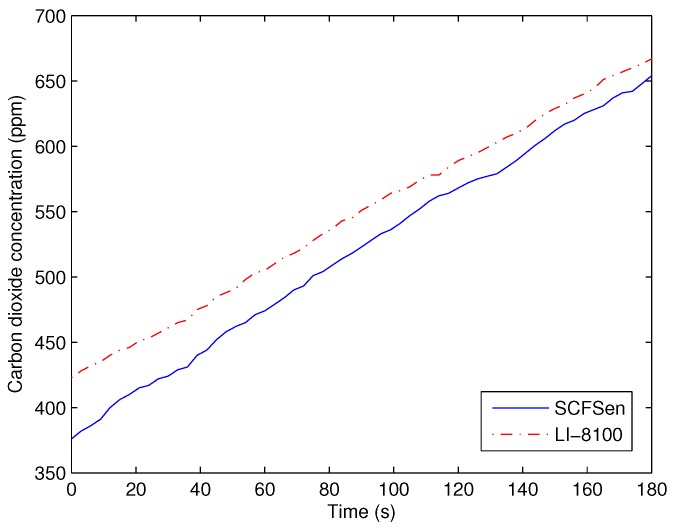
Changing of carbon dioxide concentration.

To explore the methods of calibration, we tried to find the correlation between the readings of *SCFSen* and those of other instruments such as LI-8100. At first, we measured the change of carbon dioxide concentration in the same place using both *SCFSen* and LI-8100 for adjacent 180 s, respectively, and the results in an experimental position are shown in Figure 6.

As can be seen in Figure 6 that there is a difference of almost forty ppm between the first measurements of the two instruments, which is caused by the absolute error of carbon dioxide concentration values for using different sensors. However, the differences between corresponding readings of *SCFSen* and those of LI-8100 are not a constant. We cannot calibrate the readings by simply giving a value compensation.

On the other hand, the results of the two instruments are both nearly linear, and the situations of the measuring results in other experimental positions are similar to that in this position. The reason is that the carbon dioxide concentration is increasing along with the releasing of carbon oxide from soil and the trend is as Figure 3 shown. The nearly linear property of carbon dioxide concentration can be used to calculate the changing rate of carbon dioxide concentration. According to Equation (11), the absolute error of measurements does not affect the measurement of soil carbon flux, because the measurement accuracy of soil carbon flux is mainly in relation to the changing rate other than the absolute value of carbon dioxide concentration.

The changing rates of carbon dioxide concentration measured using them are also different, which is caused by the different designing parameters of these two kinds of instruments. However, the carbon dioxide concentration measured using both of the two instruments are all nearly linear with time in the first three minutes. Therefore, we adopt the measurements in the beginning three minutes as the data to calculate the changing rate of concentration of carbon dioxide, which is the value of $\frac{\partial c_{c}^{\prime }}{\partial t}$ in Equation (11).

Because the nearly linear property of the measurement in the front three minutes, the changing rate of carbon dioxide concentration can be regarded as a constant value when using *SCFSen*, which is used for the calculation of soil carbon flux.

If the error of changing rate of carbon dioxide concentration could be eliminated, the measurement of soil carbon flux will be correct. So we intend to carry out calibration of changing rate of carbon dioxide concentration other than the individual measurements of carbon dioxide concentration. The increment of carbon dioxide concentration is caused by the releasing of carbon dioxide from soil, and the changing rate means the increment of carbon dioxide concentration in unit time. Then an intuitive hypothesis comes into our mind: The difference of sensitivities of different carbon oxide sensors leads to different changing rates in the same time zone, and the changing rates are related to the sensitivity of sensors.

### 4.2. Method of Calibration

To calibrate the changing rate of carbon dioxide concentration measured by *SCFSen* instruments, we carried out some measurements in *m* different positions using *nSCFSen* instruments and one reference instrument such as a LI-8100 or a calibrated *SCFSen*. Let $S_{0}$ denotes the reference instrument and $S_{1},S_{2},\ldots ,S_{n}$ denote the *n* randomly selected *SCFSen* instruments, $P_{1},P_{2},\ldots ,P_{m}$ denote the *m* different locations, respectively. $R_{ij}\left(0\;\;\leq \;\;i\;\;\leq \;\;n,1\;\;\leq \;\;j\;\;\leq \;\;m\right)$ denotes the changing rate of carbon dioxide concentration in three minutes using instrument $S_{i}$ in the position $P_{j}$. After calculating and analyzing the Pearson product-moment correlation coefficients between $S_{i}\left(i>0\right)$ and $S_{0}$ using Equation (18), we found that the changing rates of carbon dioxide measured using *SCFSen* are nearly linearly correlated with each other and to the results using LI-8100. More details are described in Section 5.

(18) $$r_{i}=\frac{m\sum_{j=1}^{m}R_{ij}R_{0j}-\sum_{j=1}^{m}R_{ij}\sum_{j=1}^{m}R_{0j}}{\sqrt{m\sum_{j=1}^{m}R_{ij}^{2}-{\left(\sum_{j=1}^{m}R_{ij}\right)}^{2}}\sqrt{m\sum_{j=1}^{m}R_{0j}^{2}-{\left(\sum_{j=1}^{m}R_{0j}\right)}^{2}}}$$

Thus we can calibrate *SCFSen* by linearly transforming the changing rate according to the result measured by LI-8100 or other similar devices as reference. If we can know the calibration coefficients $a_{i}$ and $b_{i}$ for a *SCFSen* instrument $S_{i}$, the changing rate of carbon dioxide concerntration measured by it, $R_{ij}$, can be calibrated to $R_{ij}^{\prime }$ using Equation (19).

(19) $$R_{ij}^{\prime }=a_{i}R_{ij}+b_{i}$$

Now we focus on the method of finding the calibration coefficients $a_{i}$ and $b_{i}$ for a *SCFSen* instrument $S_{i}\left(i>0\right)$. As the existence of measuring error, for a *SCFSen*$S_{i}$, we cannot make every calibrated value $R_{ij}^{\prime }$ equal to its reference value $R_{0j}$ in all *m* positions using the same calibration coefficients $a_{i}$ and $b_{i}$. We can only consider about the total calibration performance for all *m* positions. Here we set the requirement as the expectation of $R_{ij}\left(i>0\right)$ equaling to that of $R_{0}j$, as Equation (20).

(20) $$\frac{1}{n}\sum_{j=1}^{m}R_{ij}^{\prime }=\frac{1}{n}\sum_{j=1}^{m}R_{0j}$$

Furthermore, $a_{i}$ and $b_{i}$ should try to minimize the deviation of $R_{ij}^{\prime }$ to $R_{0j}$, as Equation (21) shows.

(21) $$\begin{array}{cc} \left(a_{i},b_{i}\right) & =arg\;min\sum_{j=1}^{m}{\left(R_{ij}^{\prime }-R_{0j}\right)}^{2}\\ & =arg\;min\sum_{j=1}^{m}{\left(\left(a_{i}R_{ij}+b_{i}\right)-R_{0j}\right)}^{2} \end{array}$$

Solve the three Equations (19), (20) and (21), and the value of $a_{i}$ and $b_{i}$ can be reached as Equation (22).

(22) $$\begin{array}{cc} a_{i} & =\frac{\sum_{j=1}^{m}R_{ij}\sum_{j=1}^{m}R_{0j}-m\sum_{j=1}^{m}R_{ij}R_{0j}}{{\left(\sum_{j=1}^{m}R_{ij}\right)}^{2}-m\sum_{j=1}^{m}R_{ij}^{2}},\\ b_{i} & =\frac{\sum_{j=1}^{m}R_{0j}-a_{i}\sum_{j=1}^{m}R_{ij}}{m} \end{array}$$

Once the calibration coefficients *a* and *b* for a *SCFSen* instrument are determined according to the mentioned method, we will use them directly in the calculation of soil carbon flux to calibrate its results. Of course the calibration coefficients for a *SCFSen* instrument depend on the parameter *m* and the features of the *m* positions during the calibration procedure. The bigger *m* is, and the wider range the features of the *m* positions include, the more accurate the calibration coefficients *a* and *b* can be reached, and the closer the calibrated measurements are to the real situation.

## 5. Experiment and Analysis

### 5.1. Calibration

**Figure 7:**
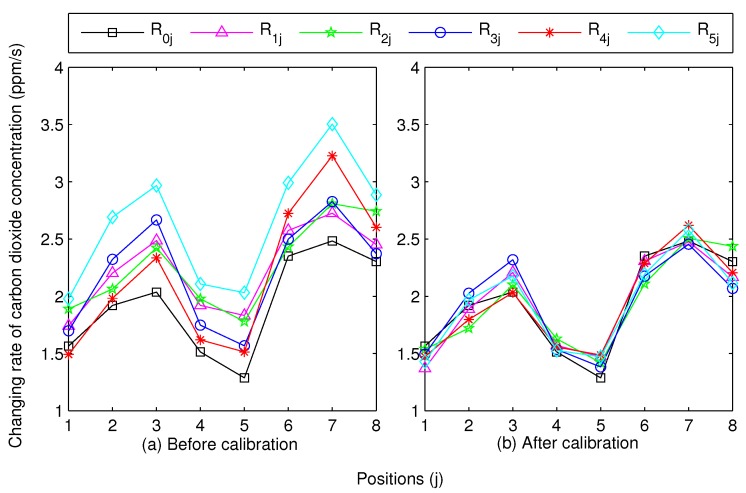
Changing rates of carbon dioxide concentration using 5 *SCFSen* nodes and a reference in 8 different positions.

In our experiment, we used 5 randomly selected *SCFSen* instruments and a reference instrument (LI-8100) in 8 different positions, which means that *n* equals 5 and *m* equals 8 in the equations in Section 4.2. The measured changing rates of carbon dioxide concentration using these instruments in these positions, $R_{ij}\left(0\;\;\leq \;\;i\;\;\leq \;\;5,1\;\;\leq \;\;j\;\;\leq \;\;8\right)$, are shown in Figure 7a.

Then we applied calibration to the measurement results according to the method in Section 4. The coefficients during the calibration are shown in Table 3. The column $r_{i}$ denotes the correlation coefficient between $S_{i}\left(1\;\;\leq \;\;i\;\;\leq \;\;5\right)$ and $S_{0}$, and the $a_{i}$ and $b_{i}$ denote the calibration coefficients of $S_{i}\left(1\;\;\leq \;\;i\;\;\leq \;\;5\right)$. As we can see that the values of $r_{i}$ are all above 0.9, which means the linear correlation between $R_{ij}\left(1\;\;\leq \;\;i\;\;\leq \;\;5\right)$ and $R_{0j}$. Using the calibration coefficients $a_{i}$ and $b_{i}$, the calibrated changing rates of carbon dioxide concentration of $R_{ij}\left(1\;\;\leq \;\;i\;\;\leq \;\;5\right)$, $R_{ij}^{\prime }$, can be get, which are shown in Figure 7b. As we can see that the calibrated results of different *SCFSen* instruments are all stay nearly accordant with the result of the reference instrument in all experimental positions after calibration.

**Figure 8:**
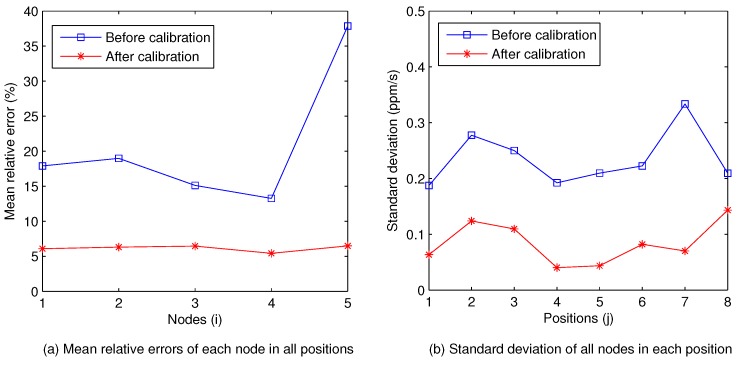
Performance of calibration.

The performance of calibration is shown in Figure 8. As we can see from Figure 8a, the mean relative errors of every *SCFSen* node in these positions are all almost 15% before calibration, and decrease to near 6% after calibration. Because there is no real soil carbon flux data, the results of LI-8100 in every position are used as the ground truths in our analysis. The difference between the results from two different instruments is reasonable, and can be eliminated by appropriate conversion if the user would like to.

Besides, the standard deviations of the changing rates measured using the 5 *SCFSen*s in each position before and after calibration are shown in Figure 8b. Obviously, the calibration operation makes standard deviations smaller in all positions, which means the stability of *SCFSen* after calibration. Here the situation in the 7th Position is a little interesting, where the standard deviation before calibration is the biggest while that after calibration becomes much smaller, which is an occasional case for the calibration coefficients are very suitable for the measurements in this position, as can be easily understood if we analyze the data in Figure 7.

There are mainly two aspects of reasons for a calibrated *SCFSen* result to be different from the result of the reference instrument at the same position. The first one is the temporal variation of soil carbon flux. Because we cannot take the measurements using a *SCFSen* instrument and a reference instrument in a position at the same time, the real values may also be different. During the calibration procedure, we can only try to minimize the influence of temporal variation of soil carbon flux by doing the measurements using different instruments with small intervals. The second reason is that the count and the features of positions selected during the calibration procedure, which may also have influence on the values of calibration coefficients and the calibrated results as mentioned in Section 4.2.

To obtain good calibration performance during the practical application, we should try to minimize the influence of these two aspects. As for the first one, the calibration operation prefers to be done in the period when the environmental parameters, such as soil temperature, humidity and so on, are relatively stable, so as to get relatively stable changing rate of soil carbon flux using the *SCFSen* and the reference, because these parameters are the influence factors to the soil carbon flux [34,35]. As to the second aspects, calibrations should be carried out in more positions with different soil conditions.

### 5.2. Deployment and Measurement

After calibration, *SCFSen* instruments can be used as sensor nodes to construct a sensor network for the measurement and estimation of regional soil carbon flux. Deploy calibrated *SCFSen*s in an area and measurements of each sampling position can be taken synchronously and periodically, and the soil carbon flux values in every position and at different time can be obtained. Based on the sensing data from these *SCFSen* measurements, the spatial heterogeneity and temporal variation properties of the regional soil carbon flux can be examined.

In practice, when we want to monitor the soil carbon flux in an area, the first step is to determine the positions to carry out the measurements, which should be done by the ecologists based on their environment domain knowledge. Once the positions are chosen, we will deploy a *SCFSen* node in each position. Due to the selection of positions by the ecologists mainly focus on the requirements of ecological representation of the measurements data, and the requirements of networking connectivity and robust are not taken into account by the ecologists, some relaying nodes have to be added in proper locations to make the network is well connected and data of measurements can be well collected. Our previous environment monitoring project, GreenOrbs [16], is successfully deployed in the forest. We use the similar type of nodes in GreenOrbs as the relaying nodes, which is more lightweight than *SCFSen* nodes and needs less energy and lower costs.

**Figure 9:**
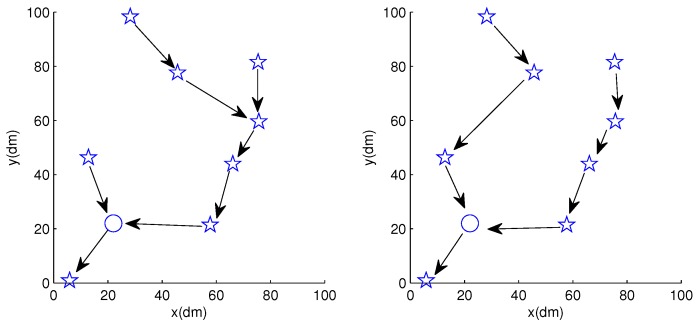
Structure of the experimental network using *SCFSen*.

Using *SCFSen* as sensor nodes in a field of 10 m × 10 m as Figure 1 shows, we carried out measurements based on the experimental sensor network. Figure 9 shows the deployment schema of the experimental sensor network, where the pentagrams denote the *SCFSen* nodes and the circle means the relaying node. The arrows between nodes demonstrate the topology of the network for two different rounds of data collections.

**Figure 10:**
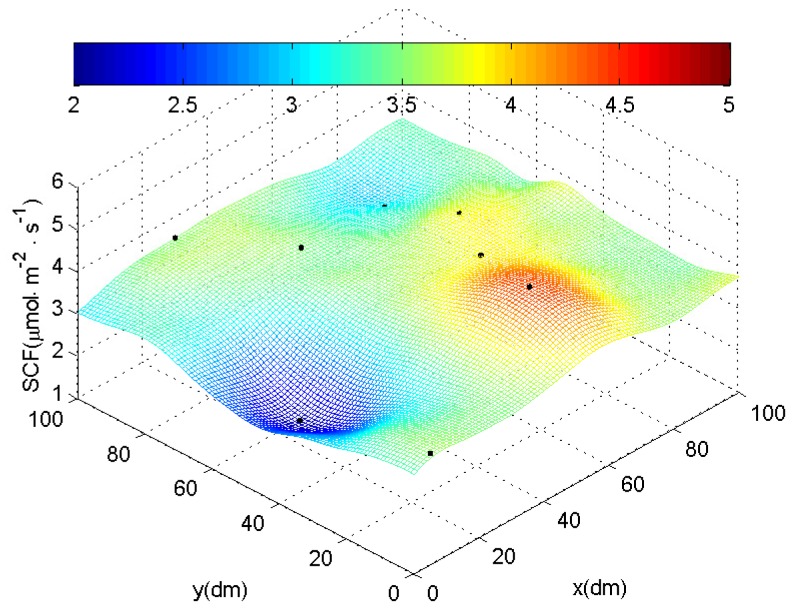
Results of the experimental measurements using *SCFSen*.

Figure 10 shows the results of the regional soil carbon flux after interpolation based on one round of measurements of these nodes using Kriging method, which is commonly used in geostatistics applications.

## 6. Conclusions

In this paper we introduced the theory, design, implementation, and calibration of an instrument, *SCFSen*, for the measurement of soil carbon flux. Because of the sensitivity and precision of the sensor chip used in *SCFSen*, the measurements have certain relative errors compared with the measurements using LI-8100, and the errors can be decreased by calibrations. It will be of benefit to the accurate estimation of regional soil carbon flux and explore the spatial heterogeneity and temporal variation properties of the regional soil carbon flux, because it can be used to measure soil carbon flux persistently in multiple positions and under synchronized control, which cannot be accomplished well using currently existing instruments. It uses a wireless transceiver chip to support wireless communication, which enable a soil carbon flux sensor network to be built if multiple such instruments in different positions are deployed. The wireless sensor network technology provides the abilities of synchronization and cooperation of multiple instruments. This instrument is of low power consumption and can keep measuring periodically, which satisfies the requirement of outside monitoring.

In the following research, we will focus on the effective nodes placing strategies for the measurement of regional soil carbon flux using *SCFSen*, which is a very challenging issue for high spatial heterogeneity and temporal variability of soil carbon flux. We intend to solve the problem via taking advantage of some domain knowledge of soil respiration, according to which the changes of soil carbon flux along with time or space possess some regularities depending on some influencing factors. Then we can fulfill the purpose of accurate measurement and estimation for regional soil carbon flux using the instrument *SCFSen*.

## Figures and Tables

**Table 1:** Chipsets adopted in *SCFSen*.

Module	Chipset
Wireless transceiver module	CC2420
Carbon dioxide sensor	T6615
Temperature and humidity sensor	SHT15
Motor drive module	ZGA17RU877i5600
Buck converter	MAX1836
Rechargeable battery	YSD-12980
LCD screen	QC12864B

**Table 2:** Energy Consumption of *SCFSen*.

Modules	Current (mA)	Duration	Illustration
Main control module	0.5	60 min	
Initialization	47.4	100 s	Warming up
Positive rotation of motor	66.4	25 s	Chamber closing
Negative rotation of motor	16.8	25 s	Chamber opening
Measurement & transmission	100.0	3 min	

**Table 3:** Correlation coefficients and calibration coefficients of *SCFSen*.

*i*	$r_{i}$	$a_{i}$	$b_{i}$
1	0.9524	1.1231	−0.5856
2	0.9417	1.0561	−0.4592
3	0.9262	0.8521	0.0468
4	0.9632	0.6618	0.4849
5	0.9456	0.753	−0.0589
